# Changes in the concentrations of biochemical indicators of diet and nutritional status of pregnant women across pregnancy trimesters in Trujillo, Peru, 2004–2005

**DOI:** 10.1186/1475-2891-12-80

**Published:** 2013-06-11

**Authors:** D Kevin Horton, Olorunfemi Adetona, Manuel Aguilar-Villalobos, Brandon E Cassidy, Christine M Pfeiffer, Rosemary L Schleicher, Kathleen L Caldwell, Larry L Needham, Stephen L Rathbun, John E Vena, Luke P Naeher

**Affiliations:** 1 The University of Georgia, College of Public Health, 150 Environmental Health Science Bldg, Athens, Georgia 30602-2102, USA; 2 Agency for Toxic Substances and Disease Registry, Atlanta, GA, USA; 3 Asociacion del Aire Ambiental, Lima, Peru; 4 National Center for Environmental Health, Centers for Disease Control and Prevention, Atlanta, Georgia, 30341, USA; 5 Department of Biostatistics and Epidemiology, The University of Georgia, College of Public Health, Paul D. Coverdell Center for Biomedical and Health Sciences, Athens, Georgia, 30602-7396, USA

**Keywords:** Micronutrients, Pregnant women, Trimester, Serum, Cord blood, Peru

## Abstract

**Background:**

In developing countries, deficiencies in essential micronutrients are common, particularly in pregnant women. Although, biochemical indicators of diet and nutrition are useful to assess nutritional status, few studies have examined such indicators throughout pregnancy in women in developing countries.

**Methods:**

The primary objective of this study was to assess the nutritional status of 78 Peruvian women throughout pregnancy for 16 different nutritional indicators including fat-soluble vitamins and carotenoids, iron-status indicators, and selenium. Venous blood samples from which serum was prepared were collected during trimesters one (n = 78), two (n = 65), three (n = 62), and at term via the umbilical cord (n = 52). Questionnaires were completed to determine the demographic characteristics of subjects. Linear mixed effects models were used to study the associations between each maternal indicator and the demographic characteristics.

**Results:**

None of the women were vitamin A and E deficient at any stage of pregnancy and only 1/62 women (1.6%) was selenium deficient during the third trimester. However, 6.4%, 44% and 64% of women had ferritin levels indicative of iron deficiency during the first, second and third trimester, respectively. Statistically significant changes (*p* ≤ 0.05) throughout pregnancy were noted for 15/16 nutritional indicators for this Peruvian cohort, with little-to-no association with demographic characteristics. Three carotenoids (beta-carotene, beta-cryptoxanthin and trans-lycopene) were significantly associated with education status, while trans-lycopene was associated with age and beta-cryptoxanthin with SES (*p* < 0.05). Concentrations of retinol, tocopherol, beta-cryptoxanthin, lutein + zeaxanthin and selenium were lower in cord serum compared with maternal serum (*p* < 0.05). Conversely, levels of iron status indicators (ferritin, transferrin saturation and iron) were higher in cord serum (*p* < 0.05).

**Conclusion:**

The increasing prevalence of iron deficiency throughout pregnancy in these Peruvian women was expected. It was surprising though not to find deficiencies in other nutrients. The results highlight the importance of continual monitoring of women throughout pregnancy for iron deficiency which could be caused by increasing fetal needs and/or inadequate iron intake as pregnancy progresses.

## Background

Vitamins and minerals, collectively referred to as micronutrients, are essential nutritional elements for all humans, especially pregnant women [1]. Pregnant women are particularly vulnerable to deficiencies in micronutrients because of the increased metabolic demands imposed by pregnancy [2]. Micronutrient deficiencies in pregnant women and women of reproductive age are recognized as major public health problems in many developing countries [3]. Maternal micronutrient deficiencies are prevalent in low-income countries mainly because of the expense of, and/or lack of access to, foods rich in multiple micronutrients [4], and could also be dependent on lack of maternal health education [5]. Access to nutrient-rich foods during pregnancy is critical because inadequate stores of micronutrients can have adverse effects on the mother, such as anemia, hypertension, complications of labor and even death [6]. The consequences to the fetus and neonate from insufficient maternal micronutrient intake include stillbirth, pre-term delivery, intrauterine growth retardation, congenital malformations, reduced immunocompetence and abnormal organ development [1].

In Peru, micronutrient deficiency has been recognized as a significant public health problem [7,8]. As an example, an estimated 42.7% of pregnant Peruvian women have anemia due to low iron intake [9]. Other studies using dietary intake patterns from nutritional surveys have demonstrated that some pregnant Peruvian women have low intakes of iodine, thiamin, folate, calcium, and zinc [7,8].

Although these studies are important, a comprehensive study describing a wide-range of biochemical indicators of diet and nutrition among pregnant Peruvian women has not been published. Biochemical indicators are compounds of potential health relevance. They are typically measured in blood or urine and are important screening tools because they are indicative of the nutritional status of an individual. Using a sample of 78 pregnant Peruvian women, the objectives of this study were to 1) quantify biochemical indicators of diet and nutrition during pregnancy through delivery, 2) assess whether select demographic characteristics (e.g., socioeconomic status [SES], education, and age) are associated with the biochemical indicators measured, 3) determine if micronutrient deficiencies exist during pregnancy, and 4) compare serum biochemical indicators of diet and nutrition for pregnant women from Trujillo, Peru with a representative sample of pregnant women from the United States. To our knowledge, this is the first study that examines micronutrient serum concentrations among pregnant Peruvian women throughout pregnancy and into delivery.

## Methods

This study was part of a larger study conducted by the University of Georgia (UGA) that was focused mainly on exposure of pregnant women in Trujillo, Peru to indoor air pollution (IAP) resulting from the residential combustion of solid biomass fuel. Pregnant women were chosen because various studies have indicated that the fetus is potentially vulnerable to IAP [10]. While contaminant-specific IAP analyses (e.g., polyaromatic hydrocarbons) for these pregnant women are presented separately [11], this paper focuses specifically on biochemical indicators of diet and nutrition using maternal and cord serum.

### Study location

This study took place between May, 2004 and February, 2005 in the La Libertad Province of Peru. The designated study area was in Trujillo, the provincial capital with a population of 757,266. An arid coastal city in northwestern Peru, Trujillo is located just west of the foothills of the Andes Mountains and is the third most populous city in Peru. Trujillo has a dry climate with an average temperature of 21°C (range: 14°C to 32°C). The residential locations of the study participants included seven different districts within 10 kilometers of the city of Trujillo: Trujillo, La Esperanza, El Porvenir, Florencia de Mora, Moche, El Milagro, and Alto Trujillo.

### Subject selection

A convenience sample of 100 non-smoking pregnant women in the first trimester of pregnancy was recruited to participate in the larger IAP study, with the goal of having enough women for sufficient power to detect differences in IAP exposures between women living in homes where gas, wood, kerosene and coal briquette were used for cooking. It was impossible to achieve balance in the number of subjects (n = 25 for each fuel category) due to time and resource constraints, and the non availability of enough women in all the fuel categories. Seventy-eight of the women ultimately gave blood for biomarker exposure analyses (e.g. biochemical indicators of diet and nutrition). All women were initially recruited as they entered/exited select health clinics throughout Trujillo. This study was approved by UGA’s Institutional Review Board with assurance ID number FWA00003901, the Centers for Disease Control and Prevention (CDC) and health authorities at the Trujillo City Hall. Informed consent was obtained from all subjects.

### Questionnaires

Questionnaires were administered to the women in Spanish during the first trimester to determine demographic characterization and exposure identification to IAP pollutants. Information was obtained on fuel type used, SES, education, age, and other demographics. It is problematic in many developing countries to use traditional measures (e.g., income) for the assessment of an individual’s financial status and SES, due to practical limitations of collecting accurate data. Most participants in this study did not report an actual income. Therefore, SES was determined mainly by assessing residential characteristics (e.g., place of residence; size of home; construction material of home; access to electricity; type of floor, toilet, water source; and fuel type used), and categorized into poor/lower SES, middle/middle SES and affluent/upper SES.

### Serum sample collection

Forty ml of antecubital venous blood were collected from each subject during every trimester of pregnancy at the Trujillo City Hall by qualified, local health professionals. Additionally, 10 ml of cord blood was collected at delivery. Clinical staffs were advised to clamp the cord immediately after delivery and one of the researchers was always available to collect the cord blood samples soon after delivery. Maternal and cord blood was drawn using 21-gauge butterfly needles and stored in Vacutainer® collection vials (Becton-Dickinson, Franklin Lakes, NJ). The blood samples were centrifuged after allowing 20–30 minutes for clotting at room temperature. Serum was then collected, transferred to multiple aliquots (0.5 ml for selenium and 1.5 ml for the other micronutrient analyses), and frozen. The samples were temporarily stored in a large refrigerator/freezer at the Trujillo City Hall until they could be transferred to a −30°C freezer where they were stored for the duration of the conduct of sample collection from May 2004 to February 2005. All frozen serum samples were then transported from Trujillo, Peru to Athens, Georgia, USA in coolers containing dry ice. Upon arrival in the US, the serum was stored at −30°C and ultimately shipped to the CDC in Atlanta, GA for laboratory analysis. Participant information was not available to CDC researchers.

### Biochemical indicators

Sixteen different biochemical indicators (retinyl palmitate, retinyl stearate, retinol, alpha-carotene, beta-carotene, cis-beta-carotene, lutein + zeaxanthin, trans-lycopene, beta-crytoxanthin, total lycopene, alpha-tocopherol, gamma-tocopherol, ferritin, iron, unbound iron binding capacity and selenium) were initially analyzed for this study. Two others, total iron binding capacity (the sum of iron and unbound iron binding capacity) and transferrin saturation (the ratio of iron to total iron binding capacity) were calculated. These particular biochemical indicators were chosen because they are typically used to assess nutritional status. In addition, retinol, alpha-tocopherol, iron levels, and selenium have been associated with adverse pregnancy/birth outcomes and/or neonatal health effects [1]. These indicators were grouped into four main classes: fat-soluble vitamins, carotenoids, iron-status indicators, and selenium. While carotenoids are fat-soluble micronutrients which are traditionally grouped under fat-soluble vitamins, they were categorized separately in this study.

Two of the 16 biochemical indicators that were measured instrumentally (i.e., retinyl palmitate and retinyl stearate) were dropped from statistical analyses because ≥40% of their values were below the limit of detection (LOD). Therefore, 16 biochemical indicators (14 analytically measured and two calculated) remained for maternal serum analyses. Similarly, seven of the 16 biochemical indicators that were measured instrumentally were dropped from the statistical analysis of cord serum concentrations (i.e., alpha-carotene, beta-carotene, cis-beta-carotene, lutein + zeaxanthin, trans-lycopene, retinyl palmitate, and retinyl stearate) because ≥40% of their values were < LOD. Therefore, 11 biochemical indicators (nine analytically measured and two calculated) were used for statistical analysis involving cord serum. The ≥40% LOD cut-off for the calculation of geometric means is a threshold used by the CDC for statistical analysis as part of the National Report on Biochemical Indicators of Diet and Nutrition in the U.S. Population 1999–2002 [12].

### Laboratory analyses

Serum concentrations of all analytes were measured at the CDC’s Nutrition Laboratory. All fat-soluble vitamins and micronutrients were measured using isocratic high performance liquid chromatography (HPLC) with multiwavelength detection. The procedure involved minor modifications of an existing published method [13]. Ferritin was measured using a turbidimetric immunoassay on the Hitachi 912 Chemistry Analyzer (Roche/Hitachi. Tina-quant® Ferritin. Package insert. 2005–11, V10). Iron and unbound iron binding capacity were measured using a FerroZine colorimetric assay on the Hitachi 912 Chemistry Analyzer (Roche/Hitachi. Fe Iron. Package insert. 2004–04, V8.). Finally, selenium was measured by using inductively coupled plasma mass spectrometry (ICP-MS) [12,14] Three levels of serum quality control samples were included in each analytical run and Westgard-type quality control multi-rules were used to judge assay performance [15]. Information on method imprecision is provided in Table 1.

**Table 1:** Biochemical indicators of diet and nutrition listed by class, laboratory analysis method, and abbreviation

**Biochemical indicator**	**Method imprecision**
**Fat-soluble vitamins (n = 5)**	
HPLC-UV^1^	
Vitamin A (retinol)	3.5-3.8% at 0.70-3.50 μmol/L
Vitamin A (retinyl palmitate)	>30% at < 0.15 μmol/L
Vitamin A (retinyl stearate)	>30% at < 0.15 μmol/L
Vitamin E (*alpha*-Tocopherol)	2.6-3.0% at 11.6-232 μmol/L
Vitamin E (*gamma*-Tocopherol)	3.0-4.0% at 2.40-11.9 μmol/L
**Carotenoids (n = 7)**	
HPLC-UV	
*alpha*-Carotene	6.0-13.0% at >0.28 μmol/L
*beta*-Carotene	6.0-13.0% at >0.28 μmol/L
*cis*-beta-Carotene	6.0-13.0% at >0.28 μmol/L
*beta*-Cryptoxanthin	6.0-13.0% at >0.27 μmol/L
Lutein + Zeaxanthin	6.0-13.0% at >0.26 μmol/L
*trans*-Lycopene	6.0-13.0% at >0.28 μmol/L
total Lycopene	6.0-13.0% at >0.28 μmol/L
**Iron indicators (n = 5)**	
Immunoturbidimetry	
Ferritin	6.1-7.4% at 79–426 μg/L
Colorimetry	
Iron	1.7-1.9% at 13.1-30.0 μmol/L
Unbound Iron-Binding Capacity	2.5-3.0% at 28.0-41.4 μmol/L
Total Iron-Binding Capacity	N/A (calculated value)
Transferrin Saturation	N/A (calculated value)
**Trace element (n = 1)**	
Atomic absorption spectrometry	
Selenium	0.04%

^1^*HPLC-UV * high performance liquid chromatography.

### Statistical analysis

Personal and household characteristics, along with the serum distributions of the biochemical indicators, are presented as descriptive statistics in Tables 2 and 3 respectively. The serum results were summarized using geometric means and their 95% confidence limits because the concentrations of the biochemical indicators were positively skewed [16].

**Table 2:** Personal and household characteristics of 78 pregnant Peruvian women

**Characteristic**	**No.**^**1**^	**%**^**2**^
**Age group (in years)**	78	---
≤ 20	16	20.5%
21–25	26	33.3%
26–29	19	24.4%
≥ 30	17	21.8%
**Education status**	78	---
Primary	16	20.5%
Secondary	45	57.7%
Superior	17	21.8%
**Socioeconomic status (SES)**	78	---
Lower	50	64.1%
Middle/Upper	28	35.9%
**Fuel type used**^**3**^	78	---
Gas	47	60.3%
No gas	31	39.7%
**Residential Location**^**4**^	78	---
Central	17	21.8%
North	56	71.8%
South	5	6.4%

^1^Total number of subjects for which the response is known.

^2^Percentages are calculated from the known number of responses in column 2.

^3^Gas = gas, combination including gas, electricity; No gas = wood, kerosene, vegetable carbon, combination not including gas.

^4^Central = Trujillo; north = La Esperanza, El Porvenir, Florencia de Mora, and El Milagro; and south = Moche and Alto Trujillo.

**Table 3:** **Serum concentration distributions of select biochemical indicators for pregnant Peruvian women**^**1**^

**Biochemical indicator**^**1**^	**LOD**^**2**^	**Trimesters 1–3 Combined**						**Cord serum**						***p*-values**^**4**^
		**% of samples below LOD**	**n**	**GM (95% CI)**^**3**^	**Median (range)**	**Min.**	**Max.**	**% of samples below LOD**	**n**	**GM (95% CI)**	**Median**	**Min.**	**Max.**	
Retinol	0.035	0.0	193	1.43 (1.40-1.49)	1.46	0.74	2.63	0.0	52	0.64 (0.60-0.69)	0.66	0.34	1.29	≤ 0.0001
Retinyl palmitate	0.025	65.3	193	NC^5^	LOD	LOD	0.11	100.0	52	NC	NC	LOD	LOD	NC
Retinyl stearate	0.013	97.4	193	NC	LOD	LOD	0.027	100.0	52	NC	NC	LOD	LOD	NC
Alpha-tocopherol	0.94	0.0	193	27.2 (26.2-28.3)	27.9	14.3	46.0	0.0	52	5.92 (5.52-6.35)	5.80	3.60	15.5	≤ 0.0001
Gamma-tocopherol	0.26	0.0	193	3.14 (2.98-3.32)	3.21	1.10	7.68	17.3	52	0.35 (0.31-0.39)	0.34	0.18	0.98	≤ 0.0001
Alpha-carotene	0.013	0.0	193	0.19 (0.18-0.21)	0.19	0.042	1.14	65.4	52	NC	NC	LOD	0.007	NC
Beta-carotene	0.015	0.0	193	0.21 (0.20-0.23)	0.21	0.050	1.90	69.2	52	NC	NC	LOD	0.005	NC
Cis-beta-carotene^6^	0.013	23.9	71	0.018 (0.017-0.020)	0.017	0.009	0.12	100.0	31	NC	NC	LOD	LOD	NC
Beta-cryptoxanthin	0.016	0.0	193	0.43 (0.39-0.47)	0.45	0.07	2.39	0.0	52	0.052 (0.045-0.063)	0.051	0.018	0.26	≤ 0.0001
Lutein + zeaxanthin	0.042	0.0	193	0.53 (0.50-0.58)	0.53	0.11	1.24	0.0	52	0.11 (0.10-0.12)	0.10	0.065	0.28	≤ 0.0001
Trans-lycopene	0.015	0.5	193	0.058 (0.052-0.063)	0.054	0.011	0.53	100.0	52	NC	NC	LOD	LOD	NC
Total lycopene	0.019	0.0	193	0.15 (0.14-0.18)	0.15	0.028	1.04	94.2	52	NC	NC	LOD	0.050	NC
Ferritin^7^	6.81	0.0	192	52.1 (45.2-60.2)	52.8	6.74	528	0.0	47	321 (272–377)	315	101	1090	≤ 0.0001
Iron	0.90	0.0	202	12.1 (11.2-13.0)	12.8	2.33	30.6	0.0	47	26.2 (24.4-28.2)	26.9	12.0	36.2	≤ 0.0001
Unbound iron capacity	1.80	0.0	182	55.6 (52.9-58.4)	56.9	24.2	128	0.0	42	10.0 (8.09-12.6)	10.4	1.79	31.7	≤ 0.0001
Total iron bound capacity^8^	N/A	0.0	181	71.3 (69.0-71.3)	70.0	34.7	134	0.0	42	38.2 (36.2-40.3)	37.5	25.1	57.7	≤ 0.0001
Transferrin saturation^9^	N/A	0.0	181	17.9 (16.4–19.6)	19.0	3.0	53.0	0.0	42	67.0 (61.7–72.6)	73.5	34.0	95.0	≤ 0.0001
Selenium	0.063	0.0	204	1.44 (1.41-1.44)	1.46	0.79	2.15	0.0	44	0.86 (0.83-0.89)	0.87	0.61	1.14	≤ 0.0001

^1^All values expressed in μmol/L unless otherwise noted.

^2^*LOD* limit of detection.

^3^*GM* geometric means; 95% CI = 95% confidence interval.

^4^*p*-values are based on a *t*-test and compare trimesters 1–3 combined with cord serum.

^5^*NC* not calculated. No calculations were conducted on samples for which 40% or more of the values were < LOD as per CDC methodology guidelines (17). For all other samples < LOD, calculations were based upon the LOD values divided by the square root of two.

^6^Because of laboratory quality control issues, the total sample size for CBC is lower compared with the remaining biochemical indicators.

^7^Values expressed in pmol/L (1 pmol/L = 2.247 ng/ml).

^8^*TIBC* total iron-binding capacity. TIBC = IRN + UIBC.

^9^Transferrin saturation = IRN/TIBC. Values expressed in %. TS does not have a specified LOD.

Concentrations of the 16 maternal and 11 cord serum biochemical indicators that were less than the limit of detection (< LOD) were imputed using the value of the LOD divided by the square root of two [17]. The Peruvian maternal (trimesters 1–3 combined) and cord serum biochemical indicators were compared using a *t*-test.

Mixed effects models, accounting for the repeated measures design, were fit to the data and used to evaluate the association between demographic characteristics and biochemical indicator analytes (i.e., the dependent variables). Consequently, a random subject effect was included in the models. The independent variables included trimester (first, second, third, and at term [i.e., via cord serum]), SES (lower and middle/upper), educational status (primary, secondary, and superior), fuel type used (gas vs. non-gas, where gas = gas, combination including gas, and electricity; no gas = wood, kerosene, vegetable carbon, and combination not including gas), age-groups (≤ 20, 21–25, 26–29, and ≥30 years old) and residential location (central = Trujillo; north = La Esperanza, El Porvenir, Florencia de Mora, and El Milagro; and south = Moche and Alto Trujillo) as categorical variables. Unless noted otherwise, *p*-values ≤ 0.05 were considered statistically significant.

All data were analyzed using SAS software, version 9.1 (SAS Institute Inc., Cary NC).

## Results

### Personal and household characteristics

Table 2 describes personal and household characteristics for the 78 women in this study. Pregnant adolescents (< 18 years) were included in the convenience sample since they are not so uncommon among the study population. Nutritional requirements of adolescents are different from those of adults. However, results were not different when adolescents were excluded from statistical analyses, and so results are presented for all subjects. Age was also controlled for in the final regression models.

The personal and household characteristics of the participants in this study appear to be consistent with Peruvian national reference sources. The median age at first birth for Peruvian mothers is approximately 23 years old [18]. While the questionnaire for this study did not specifically ask whether or not this was the participants’ first child, the median age of women in this study was 25 years old (the mean was 26; range: 14–46 years). The percentage of females with at least a secondary education in urban areas is 57.0% [19]. In the location of this study, which is an urban area, 57.7% of the participants had at least a secondary education. An estimated 33.0% of the Peruvian population uses solid fuels for cooking and heating (i.e., non gas sources such as wood, brush, charcoal briquettes) [20]. In this study, 39.7% of the participants used non-gas sources. SES for this study population could not be easily compared to the Peruvian referent population because of the method used to assess SES for the study participants. Fifty (64.1%) of the women were in a lower SES according to the categorization used for this study. Finally, most of the participants (n = 56; 71.8%) lived in districts north of Trujillo.

### Sample detection

Table 3 presents the unadjusted geometric mean serum concentrations of the biochemical indicators analyzed. Of the 14 maternal biochemical indicators (trimesters 1–3 combined) that were detected in >40% of the samples, all but one was detected in 100.0% of the women (cis-beta-carotene was detected in 76.1%). Similarly, of the 9 cord serum biochemical indicators that were detected in >40% of the samples, all but one was detected in 100.0% of specimens (gamma-tocopherol was detected in 82.7%).

### Cord serum Vs. Maternal serum concentrations

Unadjusted geometric means for the 11 cord serum biochemical indicators were compared with maternal (trimesters 1–3, combined) geometric means (Table 3). Cord serum geometric mean concentrations were significantly lower (*P* ≤ 0.0001) than maternal concentrations for eight of the 11 biochemical indicators (fat soluble vitamins–retinol, alpha-tocopherol and gamma-tocopherol; carotenoids–beta-crytoxanthin and lutein + zeaxanthin; iron-status indicators–unbound and total iron binding capacities; and selenium). Cord serum concentrations of these indicators were 1.7–9.0 times lower than the maternal concentrations (fat soluble vitamins were 2.2–9.0 times lower; carotenoids were 4.8–8.1 times lower; iron-status indicators were 1.9–5.5 times lower; and selenium was 1.7 times lower than maternal concentration). Conversely, the remaining three biochemical indicators, all of which were iron-status indicators (ferritin, iron and transferrin saturation), had significantly higher (*P* ≤ 0.0001) cord serum concentrations in comparison to maternal concentrations. For these three, cord serum concentrations were 2.2–6.2 times higher than maternal concentrations.

### Maternal biochemical indicators across trimesters

Unadjusted serum concentrations for 16 of the 18 maternal biochemical indicators (except retinyl stearate and retinyl palmitate which were detected in < 40% of the samples) were analyzed across trimesters 1–3 (Table 4). Statistically significant changes during any point in pregnancy (i.e., from trimester 1–2, 2–3, 1–3) were noted for 15/16 (93.8%) biochemical indicators (only *beta*-cryptoxanthin did not significantly change). Two of the three fat soluble vitamins increased (vitamin E as *alpha*-tocopherol [+54.1%] and *gamma*-tocopherol [30.5%]) and one decreased (vitamin A as retinol [−0.7%]) from the first to the third trimester. With the exception of cis-beta-carotene, all of the carotenoids increased from the first to the third trimester by +11.7% to +72.0%. Percentage change for cis-beta-carotene could not be calculated because of missing trimester one values. Percentage changes from the first to the third trimester were variable for the iron-status indicators. Increases were noted for unbound iron-binding capacity (+73.0%) and total iron-binding capacity (+38.1%), while decreases were noted for iron (−47.4%), ferritin (−80.3%), and transferrin saturation (−59.4%). Selenium decreased −9.8% from the first to the third trimester.

**Table 4:** **Unadjusted serum concentrations of select biochemical indicators for pregnant Peruvian women across trimesters**^**1**^

**Biochemical indicator**^**2,3**^	**Trimester 1**		**Trimester 2**		**Trimester 3**		***p*-value**^**5**^
	**n**	**GM (95% CI)**^**4**^	**n**	**GM (95% CI)**	**n**	**GM (95% CI)**	
Retinol	66	1.40 (1.34-1.46)^a^	65	1.54 (1.46-1.63)^b^	62	1.39 (1.31-1.47)^a^	0.001
Alpha-tocopherol	66	21.3 (20.3-22.5)^a^	65	29.0 (27.5-30.6)^b^	62	32.9 (31.4-34.5)^c^	≤ 0.0001
Gamma-tocopherol	66	2.67 (2.44-2.92)^a^	65	3.35 (3.06-3.68)^b^	62	3.49 (3.17-3.83)^b^	≤ 0.0001
Alpha-carotene	66	0.18 (0.16-0.20)^a^	65	0.19 (0.17-0.21)^a^	62	0.22 (0.19-0.25)^b^	0.024
Beta-carotene	66	0.20 (0.18-0.23)^a^	65	0.19 (0.17-0.23)^a^	62	0.25 (0.21-0.30)^b^	0.014
Cis-beta-carotene	0	NC^6^	53	0.017 (0.013-0.019)^a^	18	0.028 (0.020-0.037)^b^	0.01
Beta-cryptoxanthin	66	0.41 (0.35-0.49)^a^	65	0.40 (0.34-0.47)^a^	62	0.46 (0.40-0.54)^a^	0.31
Lutein + zeaxanthin	66	0.41 (0.37-0.45)^a^	65	0.58 (0.52-0.63)^b^	62	0.61 (0.57-0.67)^b^	≤ 0.0001
Trans-lycopene	66	0.047 (0.041-0.054)^a^	65	0.052 (0.045-0.061)^a^	62	0.080 (0.065-0.095)^b^	≤ 0.0001
Total-lycopene	66	0.14 (0.12-0.16)^a^	65	0.13 (0.11-0.15)^a^	62	0.20 (0.18-0.24)^b^	≤ 0.0001
Ferritin^7^	78	102 (100–141)^a^	61	37.9 (31.3-45.8)^b^	53	23.4 (19.5-27.9)^c^	≤ 0.0001
Iron	78	16.1 (14.6-17.8)^a^	64	11.8 (10.5-13.2)^b^	60	8.47 (7.43-9.71)^c^	≤ 0.0001
Unbound iron binding capacity	78	42.5 (40.1-45.1)^a^	63	64.6 (61.2-68.3)^b^	41	73.5 (67.9-79.5)^c^	≤ 0.0001
Total iron binding capacity^8^	78	60.8 (58.9-62.7)^a^	63	78.2 (75.2-81.4)^b^	40	83.9 (77.9-90.4)^b^	≤ 0.0001
Transferrin saturation^9^	78	26.6 (20.0–30.0)^a^	63	15.2 (10.0–20.0)^b^	40	10.8 (10.0–15.0)^b^	0.0001
Selenium^7^	78	1.51 (1.48-1.55)^a^	64	1.42 (1.37-1.48)^b^	62	1.37 (1.30-1.43)^b^	0.0003

^1^Does not include cord serum.

^2^Retinyl palmitate and retinyl stearate were excluded because ≥ 40% of their respective values were < LOD.

^3^All values expressed in μmol/L unless otherwise noted.

^4^*GM* geometric means; 95% CI = 95% confidence interval.

^5^*p*-values were calculated using a Mixed Effects Model procedure.

^6^*NC* not calculated. Because of laboratory quality control issues, Trimester 1 samples were not analyzed. Therefore, the sample sizes across trimesters are lower for CBC compared with the remaining biochemical indicators.

^7^Values expressed in pmol/L (1 pmol/L = 2.247 ng/ml).

^8^*TIBC* total iron-binding capacity. TIBC = IRN + UIBC.

^9^Transferrin saturation = IRN/TIBC. Values expressed in %.

Note: the superscript letters “a”, “b” and “c” are used to distinguish whether trimesters have statistically significantly (p < 0.05) different biomarker concentrations. Trimesters with different letter superscript have significantly different concentrations while those with the same superscript letters do not.

### Demographic characteristic associations

Repeated measures analysis of variance, using linear mixed effects models was used to evaluate the associations between the demographic characteristics (i.e., age group, education, SES, fuel type use, and residential location) and the serum levels of the biochemical indicators of diet and nutrition. Table 5 presents the significant maternal biochemical indicators of study participants by demographic characteristics. Only three carotenoids (i.e., beta-carotene, beta-crytoxanthin and trans-lycopene) were significantly associated with any of the demographic characteristics. Education status was significantly associated with all the three. Additionally, trans-lycopene was significantly associated with age-group, while beta-crytoxanthin was significantly associated with SES. In general, these nutritional concentrations rose with increasing age, education status, and SES. Fuel type and residential location were not significantly associated with the serum levels of any of the biochemical indicators.

**Table 5:** **Serum biochemical indicators of pregnant Peruvian women by significant covariates**^**1-2**^

**Covariate**^**3**^	**Biochemical indicator parameter estimates**		
	**Total lycopene**		
**Age group**	**GM**^**4**^	**95% CI**^**5**^	***p*-value**^**6**^
≤ 20	0.060	0.045-0.082	0.02
21–25	0.054	0.041-0.071	
26–29	0.078	0.060-0.10	
≥ 30	0.058	0.047-0.075	
	**Beta-carotene**		
**Education status**	**GM**	**95% CI**	***p*-value**
Primary	0.16	0.12-0.20	0.03
Secondary	0.24	0.20-0.28	
Superior	0.26	0.20-0.34	
	**Beta-cryptoxanthin**		
**Education status**	**GM**	**95% CI**	***p*-value**
Primary	0.33	0.26-0.42	0.02
Secondary	0.45	0.38-0.54	
Superior	0.56	0.43-0.73	
	**Total lycopene**		
**Education status**	**GM**	**95% CI**	***p*-value**
Primary	0.045	0.036-0.058	0.03
Secondary	0.063	0.052-0.076	
Superior	0.071	0.052-0.093	
	**Beta-cryptoxanthin**		
**SES**	**GM**	**95% CI**	***p*-value**
Lower	0.49	0.43-0.57	0.05
Middle/Upper	0.59	0.51-0.69	

^1^Does not include cord serum.

^2^All values expressed in μmol/L unless otherwise noted.

^3^Only covariates that significantly impact biochemical indicators are presented.

^4^*GM* geometric means.

^5^95% CI = 95% confidence intervals.

^6^*p*-values were calculated using a Mixed Effects Model procedure.

### Peruvian versus NHANES biochemical indicators

The unadjusted Peruvian maternal (trimesters 1–3, combined) geometric means are presented together with the unadjusted geometric means from a sample of pregnant women from the 2003–04 NHANES in Table 6 for comparison purposes. Geometric means are not calculated for unbound iron binding capacity and selenium for NHANES due to lack of data or too few data to provide stable estimates of geometric means, respectively. Concentrations of four biochemical indicators (i.e., a fat soluble vitamin–retinol and three carotenoids–alpha-carotene, beta-crytoxanthin and lutein + zeaxanthin) were higher among the Peruvian women compared with pregnant women in NHANES. Conversely, concentrations of seven biochemical indicators (i.e., two fat soluble vitamins – alpha- and gamma-tocopherol; three carotenoids–beta-carotene, trans-lycopene and total lycopene; and two iron-status indicators–iron and total iron binding capacity) were lower among the Peruvian pregnant women.

**Table 6:** Concentrations of biochemical indicators in Peruvian women and in US women in the 2003–04 National Health and Nutrition Examination Survey (NHANES)

	**Peru (Trimester 1–3)**^**2**^		**NHANES**	
**Biochemical indicator**^**1**^	**Peru GM (95% CI)**	**n**	**US GM (95% CI)**	**n**
Retinol	1.43 (1.40-1.49)	193	1.33 (1.28-1.38)	248
Alpha-tocopherol	27.2 (26.2-28.3)	193	29.4 (28.0-30.9)	248
Gamma-tocopherol	3.14 (2.98-3.32)	193	4.77 (4.51-5.04)	248
Alpha-carotene	0.19 (0.18-0.21)	193	0.048 (0.043-0.054)	248
Beta-carotene	0.21 (0.20-0.23)	193	0.23 (0.21-0.25)	248
Cis-beta-carotene	0.018 (0.017-0.020)	71	0.019 (0.019-0.020)	248
Beta-cryptoxanthin	0.43 (0.39-0.47)	193	0.19 (0.17-0.20)	248
Lutein + zeaxanthin	0.53 (0.50-0.58)	193	0.30 (0.28-0.31)	248
Trans-lycopene	0.058 (0.052-0.063)	193	0.41 (0.39-0.43)	248
Total lycopene	0.15 (0.14-0.18)	193	0.77 (0.72-0.81)	248
Iron	12.1 (11.2-13.0)	202	13.5 (12.7-14.3)	251
Ferritin (pmol/L)^3^	52.1 (45.2-60.2)	192	52.1 (47.0-58.0)	250
Total iron bound capacity	71.2 (69.0-71.3)	181	77.1 (75.1-79.0)	251
Transferrin saturation (%)	17.9 (16.4-19.6)	181	17.5 (16.3-18.7)	251

^1^All concentrations expressed as μmol/L except otherwise stated.

^2^The Peru concentrations are geometric means calculated from data for all pregnant women having measurements across all trimesters.

^3^For ferritin, (1 pmol/L = 2.247 ng/ml).

## Discussion

This is the first study known to examine biochemical indicators of diet and nutrition concentrations throughout the gestation period for a sample of women from Peru. While serum concentrations from two fat soluble vitamins increased overall (alpha- and gamma-tocopherol), retinol concentrations decreased slightly from the first to the third trimesters. These changes are similar to results of other studies in that retinol concentration declines slightly during gestation [21], while alpha-tocopherol concentration increases significantly [22]. Retinol concentration declines gradually in pregnancy because of hemodilution [23], while alpha-tocopherol concentration is known to increase during gestation, probably because of the hyperlipidemic state associated with pregnancy [24]. The lack of a difference in the alpha-tocopherol:cholesterol ratio across pregnancy trimesters (for first, second and third trimesters: 5.57 ± 0.36, 5.60 ± 0.37 and 5.67 ± 0.39 respectively; *P* = 0.79) among the study participants seems to corroborate this hypothesis.

All seven maternal carotenoids that were analyzed in this study increased during gestation (six increased significantly from the first to the third trimester), a result consistent with other studies [25]. Fruits and vegetables provide most of the carotenoids in the human diet, though smaller amounts come from poultry products (e.g., egg yolks) and seafood [26]. While this study did not collect detailed dietary intake histories, results from one study indicated that Peruvian women commonly consume carotenoid-rich vegetables during pregnancy with consumption increasing across pregnancy [27]. This potentially could explain the overall increase of serum carotenoid levels throughout pregnancy for participants in this study, but is at odds with the slight decline in serum retinol, which is in part derived from pro-vitamin A carotenoids, and requires further study.

The increases across trimesters in unbound and total iron binding capacities, and decreases in iron, ferritin and transferrin saturation appear to follow observations of iron status indicators and are likely due to hemodilution and an increase in erythropoiesis during pregnancy [28]. The decrease in selenium is also consistent with findings in other studies [29]. Physiological dilution during the last trimesters of pregnancy likely results in a decrease of serum selenium concentrations [29].

Concentrations of alpha-tocopherol were higher than gamma-tocopherol among the pregnant women in this study. While gamma-tocopherol tends to be the most abundant form of vitamin E in the diet, serum levels are usually lower than alpha-tocopherol [30]. This is probably because the bioavailability of gamma-tocopherol as assessed in animal studies, are lower than those of alpha-tocopherol [30]. Alpha-tocopherol transport protein in the liver binds preferentially to alpha-tocopherol [30,31].

Cord serum concentrations can be viewed as a surrogate for measuring placental transfer of micronutrients from mother to infant. Concentrations for three of the iron-status indicators (i.e., ferritin, iron and transferrin saturation) appeared to substantially increase from mother to infant among participants in this study. These results are in close agreement with previous reports [32,33]. While many questions exist concerning the mechanisms by which iron is transferred to the fetus, previous findings indicate that most of the physiologic regulation of iron transfer to the fetus occurs at the level of the gut and suggests that the iron needs of the fetus take priority over maternal requirements [34]. Such fetal needs may explain this substantial increase in iron-status indicators from mother to infant, and in combination with a possible lack of corresponding increase in maternal dietary or supplementary iron intake may be responsible for the increase in the number and percentage of iron deficient women across pregnancy.

Five of the 16 maternal biochemical indicators analyzed, retinol (20 μg/dl = 0.70 μmol/L), alpha-tocopherol (500 μg/dl = 11.6 μmol/L), ferritin (15 ng/ml = 33.7 pmol/L), selenium (70 ng/ml = 0.90 μmol/L) and transferrin saturation (16%), have accepted cut-off concentrations used to determine risk of clinical deficiency in adults (Table 7) [35-38]. The serum values for retinol, alpha-tocopherol, and selenium suggest that little-to-no deficiencies occurred among pregnant Peruvian women for these biochemical indicators. However, the percentages of the two remaining biochemical indicators (ferritin and transferrin saturation) suggest that a high percentage of the pregnant Peruvian women experienced iron-status indicator deficiencies in the second and third trimesters.

**Table 7:** **Micronutrient deficiencies among pregnant Peruvian women in comparison with pregnant women from 2003–04 NHANES**^**1-2**^

**Biochemical indicators**	**Deficient cut-off concentration**	**Peruvian pregnant women**									**NHANES pregnant women**		
		**Trimester **^**1**^			**Trimester **^**2**^			**Trimester **^**3**^					
		**Deficient**	**Total**	**%**^3^	**Deficient**	**Total**	**%**	**Deficient**	**Total**	**%**	**Deficient**	**Total**	**%**
Ferritin	≤ 15 ng/mL^4^ (33.7 pmol/L)	5	78	6.4	27	61	44.3	38	53	71.7	92	250	36.8
Selenium	≤ 70 ng/mL (0.90 μmol/L)^5^	0	78	0.0	0	64	0.0	1	62	1.6	0	2	0.0
Transferrin saturation	≤ 16%^4^	10	78	12.8	29	63	46.0	31	40	77.5	100	251	39.8

^1^Of the 16 maternal biochemical indicators analyzed for this study, only 5 have clinically deficient definitions established: retinol, α-tocopherol, ferritin, selenium, and transferrin saturation. Since none of the Peruvian women were clinically deficient in retinol and α-tocopherol, these values are omitted.

^2^Micronutrient deficiency definitions do not necessarily pertain to newborns; therefore, cord serum was not included.

^3^% = deficient women/total women × 100.

^4^(23).

^5^(24).

It seems that the Peruvian pregnant women had higher concentrations of serum vitamin A [retinol]; alpha-carotene, beta-cryptoxanthin, and lutein + zeaxanthin compared to pregnant women in the 2003–04 NHANES. This suggests that the Peruvian women were getting higher amounts of select animal (e.g., beef, liver, pork) and plant sources (e.g., carrots, sweet potatoes, pumpkin) in comparison to the US women. However, the Peruvian pregnant women appeared to have had lower concentrations of seven biochemical indicators (i.e., alpha-tocopherol, gamma-tocopherol, beta-carotene, lycopene, total lycopene, iron and total iron-binding capacity). This indicates that the pregnant Peruvian women were getting lower amounts of select plant sources (e.g., tomato and tomato products; soy and corn oils, beans, lentils) in comparison to the US women.

In general, cord serum concentrations for the four classes of biochemical indicators examined in this study tended to be within the ranges cited in other studies. As an example for the fat soluble vitamins, mean cord serum concentration of vitamin A (retinol) for this study was 0.64 ± 0.016 μmol/L (18.4 ± 4.8 μg/dL) compared with 0.55-1.20 μmol/L (15.7–34.4 μg/dL) reported for other studies [39,40]. As an example for the carotenoids, mean cord serum concentration of lutein + zeaxanthin for this study was 0.11 ± 0.034 μmol/L (6.6 ± 2.2 μg/dL) compared with 0.094-1.3 μmol/L (5.4–7.4 μg/dL) reported for other studies [41,42]. Mean cord serum total iron binding capacity concentration for this study was 38.2 ± 6.75 μmol/L (216.6 ± 37.7 μg/dL), while a range of 36.4-48.5 μmol/L (203.5-271.0 μg/dL) has been reported for other studies [43,44]. Finally, mean cord serum selenium concentration for this study was 0.86 ± 0.11 μmol/L (68.2 ± 8.3 ng/mL) compared with 0.43-1.52 μmol/L (34.3–119.9 ng/mL) reported for other studies [45,46].

Serum concentrations of beta-carotene, beta-crytoxanthin and trans-lycopene significantly rose as the education level increased. Other studies have documented similar findings showing that beta-carotene-rich foods are consumed by higher educated pregnant women versus lower educated pregnant women [47]. Concentrations of beta-crytoxanthin rose significantly with SES, while serum concentrations of trans-lycopene rose significantly with age. These findings have been reported in other studies, although, not specifically among pregnant women [48].

There are several limitations with this study. First, the sample size decreased from 78 to 62 mothers from the first to the third trimester and cord serum samples were provided by only 52 subjects. Participant attrition was due to factors such as relocation and communication issues (e.g., difficulty in contacting participants because their lack of phone access). Also, while characteristics of the participants in this study (e.g., age at first birth, educational status, fuel type used) appear to be consistent with national referent sources [18-20], these study participants are not necessarily representative of all pregnant Peruvian women since recruitment was done through convenience sampling. For example, the WHO estimates that 6.5% of Peruvian women are affected by night blindness (which is directly related to retinol deficiency) [49]. Countries where ≥5% or more of the population experiences night blindness are considered significantly deficient in retinol [50]. The results from this study showed that none of the pregnant Peruvian women were retinol deficient. Additionally, non-smoking pregnant women were recruited into this study. However, a 12.1% smoking prevalence was reported for Arequipa [51], Peru’s second largest city in a population based study that was conducted between 2004 and 2006, a period overlapping the conduct of the current study. Finally, it is unclear as to what stage of pregnancy the assays were conducted for the NHANES comparison pregnant women cohort. Nevertheless, the NHANES cohort was the most stable, representative population available for comparison purposes.

## Conclusion

Requirements for many micronutrients increase during pregnancy. Serum concentrations of biochemical indicators of diet and nutrition changed significantly throughout pregnancy and at term in the sample of pregnant Peruvian women in this study, with little associations with demographic characteristics. While this group of pregnant women appears to have adequate serum concentrations of fat soluble vitamins, carotenoids, and trace elements, many of the women had low ferritin and transferrin saturation levels, which are indicative of iron depletion and deficiency. The results of the study confirm that serum micronutrient concentrations could change during pregnancy. This is especially of importance to ferritin and transferrin saturation for which increasing number of women had levels indicative of iron deficiency from the first to the third trimester.

## Competing interests

D.K. Horton, Olorunfemi Adetona, M. Aguilar-Villalobos, B.E. Cassidy, C.M. Pfeiffer, R.L. Schleicher, K.L. Caldwell, L.L. Needham, S.L. Rathbun, J.E. Vena, and L.P. Naeher declare that they have no competing interests.

## Authors’ contributions

L.N., M.A., and B.C. designed the study; L.N., M.A., and B.C. conducted the research; K.H., O.T., L.N., S.R., and J.V. analyzed the data; C.P., R.S., K.C., and L.N. conducted the laboratory analyses; O.T. reviewed the manuscript; K.H. and L.N. wrote the paper, and K.H. had primary responsibility for the final content. All authors read and approved the final manuscript.
